# Perceived Barriers to Walking for Physical Activity

**Published:** 2006-09-15

**Authors:** Genevieve F Dunton, Margaret Schneider

**Affiliations:** Department of Psychology and Social Behavior, Institute for Health Promotion and Disease Prevention Research, Keck School of Medicine of USC. At the time this study was conducted, Dr Dunton was affiliated with the Department of Psychology and Social Behavior, University of California, Irvine, Irvine, Calif.; Department of Planning, Policy and Design, University of California, Irvine, Irvine, Calif

## Abstract

**Introduction:**

Although the health benefits of walking for physical activity have received increasing research attention, barriers specific to walking are not well understood. In this study, questions to measure barriers to walking for physical activity were developed and tested among college students. The factor structure, test–retest and internal consistency reliability, and discriminant and criterion validity of the perceived barriers were evaluated.

**Methods:**

A total of 305 undergraduate students participated. Participants had a mean age (± SD) of 20.6 (± 3.02) years, and 70.3% were female. Participants responded to a questionnaire assessing barriers specific to walking for physical activity. Perceived barriers to vigorous exercise, walking for transportation and recreation, and participation in lifestyle activities (such as taking the stairs instead of the elevator) were also assessed. Subsamples completed the walking barriers instrument a second time after 5 days in order to determine test–retest reliability (n = 104) and wore an accelerometer to measure moderate-intensity physical activity (n = 85).

**Results:**

Factor analyses confirmed the existence of three factors underlying the perceived barriers to walking questions: appearance (four items), footwear (three items), and situation (three items). Appearance and situational barriers demonstrated acceptable reliability, discriminant validity, and relations with physical activity criteria. After we controlled for barriers to vigorous exercise, appearance and situational barriers to walking explained additional variation in objectively-measured moderate physical activity.

**Conclusion:**

The prediction of walking for physical activity, especially walking that is unstructured and spontaneous, may be improved by considering appearance and situational barriers. Assessing barriers specific to walking may have important implications for interventions targeting walking as means for engaging in physical activity.

## Introduction

Over the past decade, the health benefits of moderate-intensity physical activity have received increased research attention (1). In particular, brisk walking (≥3.5 mph) has been shown to reduce body fat (2,3), lower blood pressure (2,4), increase high-density lipoprotein (2,5,6), and reduce risks of bone fracture (7). Brisk walking has also been associated with lower mortality rates from cardiovascular disease and cancer (8-10). According to the 1996 *Surgeon General's Report on Physical Activity and Health*, individuals of all ages should obtain "a minimum of 30 minutes of physical activity of moderate intensity on most, if not all days of the week" (11). Although brisk walking is a readily available activity (i.e., requires no special equipment or training) (12), it is estimated that only about 21% to 34% of U.S. adults meet public health recommendations for physical activity by walking (5 times per week for at least 30 minutes) (13,14). Therefore, understanding factors that influence walking is critical to efforts to eliminate barriers and promote moderate-intensity activity among an increasingly sedentary U.S. population (15).

Correlates of vigorous-intensity exercise have received substantial research attention (16), yet fewer studies have considered predictors of walking for physical activity. In particular, research that examines the types of barriers that prevent participation in regular walking is scarce. The prominent role of perceived barriers to physical activity and other preventive health behaviors is described in the Health Belief Model (17). When individuals perceive greater barriers to performing a particular health-protective action, they are less likely to engage in that behavior. Although barriers to vigorous-intensity physical activity such as lack of time, cost, and lack of programs are well-documented (18,19), less is known about factors that may impede walking. The lack of available information on this issue is largely attributable to instrumentation limitations. Measures designed to assess perceived barriers focus heavily on factors that hinder vigorous-intensity exercise (18). For the most part, these instruments typically do not include barriers that are unique to walking as a form of physical activity.

There are a number of reasons to suggest that barriers to walking are different from barriers to vigorous-intensity exercise. As compared to vigorous exercise, walking is a relatively accessible form of physical activity. It requires less cardiovascular and muscular effort and is less likely to cause serious injury. Walking is also inexpensive and does not require special equipment or facilities. Furthermore, walking for physical activity is often unplanned and spontaneous (e.g., taking the stairs instead of the elevator, parking farther away from destinations). It can also serve a utilitarian function, such as walking for transportation or walking to satisfy occupational responsibilities. For these reasons, commonly assessed barriers to vigorous exercise, such as feeling self-conscious, lacking energy, fear of injury, and lacking financial resources (18), may pose fewer concerns for walking. Instead, walking for physical activity may be hindered by unique factors such as concern over appearance (e.g., perspiring), clothing restrictions (e.g., uncomfortable shoes), and having to carry other items (e.g., shopping bags, books). These types of barriers do not commonly appear on instruments designed to measure vigorous exercise barriers and may play an important role in explaining walking for physical activity.   

In this study, questions to assess barriers specific to walking for physical activity were developed and tested among college students. Barriers previously generated through formative research were converted into survey questions and evaluated for their factor structure, test–retest and internal consistency reliability, and discriminant and criterion validity. Overall, the goals of this study were to 1) examine the structure and measurement properties of the perceived barriers to walking items; 2) determine whether perceived barriers to walking are conceptually distinct from perceived barriers to vigorous activity; and 3) determine whether perceived barriers to walking are related to physical activity criteria.

## Methods

### Development and description of walking barriers questionnaire

Potential barriers to walking were identified through open-ended interviews and focus groups with adolescents and young adults (ages 15–22 years). To elicit thoughts about barriers to walking, focus group participants were presented with descriptions of situations that they were likely to encounter in their everyday lives. Participants were then asked about factors that may prevent walking in those situations. Frequently-mentioned barriers were converted into scale items and pilot tested among young adults for clarity and coherence. After minor revisions and rewording, twelve items were developed for a questionnaire. These items assess the extent to which barriers — including concern over perspiring in nice clothing, fear of ruining hairstyle, fear of ruining nice clothing, wearing restrictive clothing, foot pain, blisters, uncomfortable shoes, having a lot to carry, time constraints, a lack of sidewalks, and hot and cold weather — impede walking for physical activity. To clarify the targeted level of physical activity, all items are prefaced by the following question: "Thinking about the past 3 months, how much do the following things prevent you from accumulating at least 30 minutes of walking throughout your daily routine?" Responses to items were provided using a 4-point Likert-type scale, with 1 = not at all, 2 = slightly, 3 = somewhat, and 4 = a great deal. A response of *does not apply* was also available.

### Participants

The sample was composed of 305 undergraduate students from a major university in southern California. Participants ranged in age from 18 to 46 years with a mean age (± SD) of 20.6 (± 3.0), and 70.3% were female. The sample reported their overall health to be good, with a mean (± SD) response of 3.5 (± 0.9) on a 5-point scale; average body mass index (BMI) in kg/m^2^ was 22.6 (± 4.0). BMI was calculated from self-reported height and weight. The ethnic breakdown of the sample was 49.8% Asian-American or Pacific Islander, 18.8% white, 15.8% Hispanic, and 15.6% other.

### Measures

Perceived barriers to regular vigorous-intensity physical activity were measured through an instrument developed by Allison et al (18). The instrument is a scale composed of 16 items; nine items reflect internal barriers such as lack of energy, lack of self-discipline, and feeling stressed, and seven items reflect external barriers such as time constraints and cost. The perceived barriers instrument uses a 5-point response scale from 1 (not at all) to 5 (a great deal). In this study, internal consistency for the internal barriers subscales (Cronbach α = .82) and external barriers subscales (Cronbach α = .76) was satisfactory. Although this instrument was originally designed to assess perceived barriers to vigorous exercise among high-school students, it was determined to be the most relevant instrument of its kind for college-aged students. 

A dual-mode accelerometer (Manufacturing Technology, Inc [MTI], Ft Walton Beach, Fla, model 7164) provided an objective measurement of physical activity. The device is small (5.1 x 4.1 x 1.5 cm, 14g) and collects activity data unobtrusively because it is attached to a nylon belt worn around the waist. An analog bandpass filters the instrument's acceleration signal, which is subsequently digitized by an eight-bit analog-to-digital converter at a sampling rate of 10 samples per second and stored in 1-minute intervals (20). Once these data were downloaded, a data summary program (Actisoft Analysis Software, MTI, Ft Walton Beach, Fla) calculated time (in minutes) spent in moderate activity (e.g., brisk walking, 3–6 metabolic equivalent values [METs]) using the original cut-off points (1951–5724 counts per minute) established by Freedson et al (21). To adjust for differences in total monitoring time, the percentage of total minutes spent in moderate activity was computed. Accelerometers were worn for 4 consecutive days. This monitoring period has been determined to produce results that are at least 80% reliable (22). Participants with fewer than 48 hours of total monitoring for the 4 days (i.e., 12 waking hours per day) were excluded from the analyses.

Walking for transportation and walking for recreation were measured using a 3-Day Physical Activity Recall (3DPAR) validated by Motl et al (23). Participants recalled their activity for the previous 3 days between 7:00 am and 11:30 pm, segmented into 30-minute intervals. The number of 30-minute intervals of walking for transportation and walking for recreation were counted across the 3 days. Because of the substantial number of participants who reported no walking for transportation and no walking for recreation during the 3-day period, these variables were both dichotomized into *some* or *no* walking.

Participation in lifestyle physical activities was assessed with the Stanford Usual Physical Activity Questionnaire (24). Participants reported their usual participation in six lifestyle activities using a yes–no scale. These activities included taking the stairs instead of the elevator, walking short distances instead of driving, parking away from a destination in order to walk more, walking during lunch or after dinner, getting off at a bus stop before a destination and walking, and other extra walking or stair climbing for exercise.

### Procedure 

College students volunteered for the study in exchange for course credit. A sample of 305 students completed the walking and vigorous exercise barriers instruments, 3DPAR, and Stanford Usual Physical Activity Questionnaire. Of these individuals, a random subsample of 104 participants was asked to complete the walking barriers instrument a second time after 5 days and to wear the MTI accelerometer device for the 4 intervening days. Ninety-six participants agreed to wear the accelerometer, of which 88% (n = 85) met the minimum compliance criteria. Because of the limited number of MTI monitors available, it was not feasible to obtain accelerometer data from all of the original study participants. However, the subsample did not significantly differ from the larger sample on any of the key variables of interest (e.g., perceived barriers and physical activity levels [data not shown]). The University of California, Irvine Institutional Review Board approved the procedures, and all participants provided written informed consent. Data were collected during 2003.

### Statistical analyses

Data were screened for violations of statistical assumptions (e.g., normality, linearity) before the analyses. The percentage of MTI monitoring time spent in moderate-intensity physical activity was positively skewed and thus was subjected to a square root transformation. Missing data were handled with listwise deletion for principal components analysis (PCA) and hierarchical regression and pairwise deletion for all other statistical analyses.

PCA was performed to investigate the underlying factor structure of the perceived barriers to walking items. PCA is a statistical technique used to uncover coherent and independent groups of variables within a larger set (25). These groups of variables are called factors. In PCA, variables within each factor are more highly correlated with each other than with other factors. For this study, PCA was used to understand which walking barriers should be grouped together. To determine how the variables should be combined, PCA assigns values to each variable that reflect the extent to which it is related to each factor (i.e., factor loadings). The current study used an orthogonal rotation of the factor axes (varimax) to maximize the variance of the squared loadings of a factor on all the variables. This approach tends to maximize higher factor loadings and minimize lower factor loadings to yield results that facilitate the identification of each variable with a single factor.

Two separate PCAs were conducted. The first was used to determine the factor structure of the barriers to walking instrument and to identify any items that did not contribute to the integrity of the instrument. The second, in which the items from the walking barriers instrument were subjected to PCA together with the items from the vigorous activity barriers instrument, functioned to confirm that these two instruments were, in fact, measuring distinct constructs.

Further analyses evaluated the reliability and validity of the walking barriers items. The test–retest reliabilities of the walking barriers factors were evaluated through intraclass correlations of scores from each test administration. Pearson bivariate correlations assessed the factors for discriminant validity (i.e., divergence from the vigorous exercise barriers scales). Criterion validity (i.e., convergence with physical activity indicators) was evaluated with *t* tests comparing groups of individuals indicating some vs no walking for transportation and recreation on the 3DPAR. *T* tests also compared participants reporting yes vs no to the questions on the Stanford Usual Physical Activity Questionnaire. Criterion validity was further assessed by using objective measurements of moderate-intensity physical activity (via the MTI accelerometer). A stepwise hierarchical regression was used to determine if the walking barriers factors (entered in the second step) explained unique variation in moderate-intensity physical activity (measured by MTI accelerometer) after controlling for internal and external barriers to vigorous exercise (entered in the first step).

## Results

### Descriptive statistics

On average, participants wore the accelerometer about 14.5 hours on each of the 4 days of monitoring (data not shown). During this time, they engaged in an average (± SD) of approximately 29.9 (± 17.1) minutes of moderate-intensity physical activity per day*.* On the 3DPAR, participants reported an average of 18.2 (± 31.0) minutes of walking for transportation and 8.2 (± 20.0) minutes of walking for recreation per day. In total, 47.4% of participants reported some walking for transportation, and 25.0% of participants reported some walking for recreation during the past 3 days. Among participants, 80.6% usually took the stairs instead of the elevator, 67.8% usually walked short distances instead of driving, 25.0% usually parked away from a destination in order to walk more, 24.3% usually walked during lunch or after dinner, 4.6% usually got off at a bus stop before their destination in order to walk more, and 52.0% usually performed extra walking or stair climbing for exercise.

Means and SDs for the walking barriers items are shown in Table 1. In general, the mean ratings for the barriers items were low to moderate (i.e., scores were between 1 and 2 on a 4-point response scale). Lack of time, having a lot to carry, and wearing uncomfortable shoes were rated the highest. Blisters, concern over ruining one's hairstyle, foot pain, and lack of sidewalks were considered to pose the least hindrance to walking for physical activity.

### Factor structure

In the PCA of the barriers instrument, the first iteration of the PCA found that two of the barriers to walking, hot weather and cold weather, did not significantly load onto any of the factors. Thus, these two items were removed, and the PCA was rerun. The second iteration of the PCA found that all of the walking barriers items significantly loaded onto one of three factors, which accounted for 61.96% of the variance. The rotated factor loadings of the items are shown in Table 1. Loadings greater than .50 indicated significant factor identification, as this suggests at least a 25% variance overlap between the item and the factor (26). The four items clearly loading onto the first factor were barriers related to personal appearance (i.e., perspiring, ruining nice clothing, ruining hairstyle, restrictive clothing). Three perceived barriers pertaining to footwear (i.e., uncomfortable shoes, blisters, foot pain) loaded onto the second factor. The third factor consisted of three situational barriers (i.e., lack of time, having a lot to carry, and lack of sidewalks).

In the PCA of the walking barriers and vigorous activity barriers instruments combined, the number of factors was constrained to two in order to reflect the major underlying conceptual differences between walking and vigorous exercise barriers. Results found that the 16 vigorous exercise barriers loaded significantly onto the first factor, and the 10 walking barriers items loaded onto the second factor. The majority of the factor loadings were greater than .50. There was no significant overlap in item loading between the two factors (data not shown).

### Reliability

Internal consistency and test–retest reliabilities for the walking barriers factors were acceptable. Chronbach α values were as follows: 0.82 for appearance barriers, 0.65 for footwear barriers, and 0.53 for situational barriers. Five-day test–retest reliability for the mean of the appearance barriers was 0.91 (95% CI, 0.87–0.94); for the mean of the footwear barriers, 0.71 (95% CI, 0.55–0.81); and for the mean of the situational barriers, 0.77 (95% CI, 0.65–0.85).

### Discriminant validity

Discriminant validity of the walking barriers factors was assessed through relationships with the vigorous physical activity barriers scales. Table 2 shows the intercorrelations between walking barriers and vigorous exercise barriers. The correlations between factors within each construct (e.g., appearance and footwear barriers to walking, *r* = .497; internal and external barriers to vigorous exercise, *r* = .614) were generally larger than the correlations between factors from different constructs (e.g., situational barriers to walking and internal barriers to vigorous exercise, *r* = .179; footwear barriers to walking and external barriers to vigorous exercise, *r* = .230). 

### Criterion validity

To evaluate the criterion validity of the walking barriers factors, we first examined relationships with objectively measured physical activity. Bivariate correlations showed that appearance and situational walking barriers were significantly negatively related to moderate-intensity activity as measured by MTI accelerometer (Table 2).

The association of perceived barriers to participation in lifestyle activities and walking for transportation and recreation was also examined (Table 3). Based on the Stanford Lifestyle Activity Questionnaire, appearance barriers to walking were significantly associated with taking the stairs instead of the elevator and walking instead of driving. Situational barriers to walking were related to taking the stairs instead of the elevator and walking at lunch. Similarly, situational barriers were related to walking for transportation as assessed by the 3DPAR. In contrast, walking for recreation, assessed with the 3DPAR, was associated with neither appearance barriers nor situational barriers to walking.

A hierarchical regression was conducted in order to determine if walking barriers predicted moderate activity above and beyond the effects of vigorous exercise barriers. Footwear barriers to walking were not included in the regression model because they were unrelated to physical activity criteria in bivariate analyses (Table 2). The step including the appearance and situational barriers to walking explained a significant proportion of variation in moderate-intensity physical activity after controlling for internal and external barriers to vigorous activity (change in *R* ^2^ = .121 for the second step, *P* = .01). Internal and external vigorous exercise barriers were unrelated to moderate-intensity physical activity after taking into account the effects of the walking barriers. The entire model explained 13.9% of the variance in the percentage of time spent in moderate-intensity physical activity.

## Discussion

This research tested the factor structure, reliability, and validity of questions designed to assess perceived barriers to walking for physical activity. A factor analysis revealed the presence of three underlying factors: appearance barriers (four items), footwear barriers (three items), and situational barriers (three items). In general, the factors demonstrated acceptable test–retest reliability, internal consistency, and expected relations to physical activity criteria. Footwear barriers to walking, however, were unrelated to any of the measures used to assess criterion validity. Situational and appearance barriers to walking were significantly associated with lifestyle activities and walking for transportation; they also explained additional variance in moderate-intensity physical activity that was not captured by vigorous physical activity barriers. Overall, results suggest that it may be important to consider barriers related to appearance and situational characteristics when trying to understand levels of walking for physical activity among young adults.

The PCA showed that concerns over preserving personal appearance reflect an underlying set of barriers that is distinct from barriers imposed by footwear (e.g., uncomfortable shoes) or the situation itself (e.g., lack of time, having to carry things). The fact that people who reported more appearance-related barriers engaged in lower levels of objectively measured moderate-intensity walking suggests that concerns over perspiring and ruining nice clothing play an important role in the walking decisions of young adults. It is possible, however, that the desire to preserve personal appearance serves as a greater obstacle to walking for some people than others, especially individuals who place a greater value on outward appearance or whose occupational situation demands certain appearance standards. It is also possible that the influence of appearance-related barriers depends on the purpose of the walking. Results suggest that concern over appearance is more of a factor for unstructured bouts of walking. Individuals who usually take the stairs instead of the elevator and walk short distances instead of driving perceived fewer appearance-related barriers. In contrast, appearance concerns were unrelated to lunchtime walking and walking for transportation and recreation. These findings lend support to the conclusion that the desire to preserve personal appearance plays a larger role in walking decisions that are unplanned and spontaneous, instead of those that are premeditated.

Situational barriers to walking, including lack of time, having a lot to carry, and lack of sidewalks, formed another factor that was identified by PCA. Situational barriers were significantly negatively related to moderate-intensity physical activity measured through the accelerometer. Individuals who believed that situational factors prevented their participation in walking engaged in less moderate-level activity overall. Results suggested that situational barriers interfered with walking for transportation but not walking for recreation. Perceptions of situational barriers were also lower among individuals who usually take the stairs instead of the elevator and who walk during lunch or on breaks. These results suggest that situational constraints are also more likely to impede unstructured walking than walking that is planned in advance. Individuals may encounter opportunities to walk during their daily routine but forgo these opportunities because of issues such as having a lot to carry and not having enough time.

From an intervention standpoint, these results suggest that efforts to reduce appearance barriers and situational barriers may increase levels of walking among young adults. In particular, college students may engage in more walking throughout the day if they make special arrangements such as bringing along a change of clothes for the walk to or from class or work. Walking for physical activity could be facilitated if universities and employers provided changing rooms and showers to accommodate individuals who travel to work in whole or partially by foot. To combat barriers introduced by time constraints, young adults could be encouraged to walk during activities that would otherwise be performed in a sedentary manner. For example, they could walk while talking on the phone or when visiting with friends. Young adults could also be encouraged to walk short distances instead of driving (e.g., when going from one store to another in the same parking lot). Interventions could also prompt individuals to plan ahead and bring bags or carts along to carry items such as books and groceries that may hinder walking for transportation purposes.

The fact that the prediction of moderate-intensity physical activity was significantly improved by considering walking-specific barriers raises some important methodological questions. One question raised is the appropriateness of using instruments that focus on vigorous-intensity exercise to measure correlates of physical activity when walking is the outcome of interest. Although psychosocial and environmental determinants of vigorous physical activity have been well-established in the literature (16), variables specific to walking need to be identified and incorporated into future measures. In light of recent evidence illuminating the health benefits of moderate-intensity physical activity (1), the development of instruments assessing perceived benefits, self-efficacy, social support, and enjoyment specific to walking may be a useful endeavor.

This study had a few methodological limitations. Because of the cross-sectional nature of the study design, we cannot make any inferences about the causal nature of the relationships between perceived walking barriers and walking for physical activity. Also, it is not possible to tease apart sources of physical activity with the accelerometer. Therefore, moderate physical activity assessed with the MTI could include activities other than walking (e.g., gardening, housework). Furthermore, the device is unable to identify the purpose of the activity (i.e., transportation, recreation, or occupation). However, information about walking for transportation and recreation and participation in lifestyle activities reduces some uncertainty about this issue. Another limitation is introduced by the ethnic composition of the sample (about 50% Asian-American or Pacific Islander and 20% white). This distribution may not reflect undergraduate student populations in other areas of the United States. Some caution should also be taken when generalizing the results of this study to groups of young adults not attending college or to other community samples. College students might have unique lifestyle characteristics (e.g., transportation restrictions, more flexibility in daily routines) and encounter environments (e.g., pedestrian-friendly college campuses) that are not common to working adults. Additional research is needed to test the reliability and validity of the walking barriers measure in middle-aged and older adults.

Overall, greater appearance and situational barriers were associated with lower levels of walking, especially walking that is unstructured and spontaneous. Given the large number of sedentary adults in the United States (15) and the substantial health benefits gained from becoming moderately active (26), understanding psychosocial influences on unstructured walking may have important public health implications. Intervention programs targeting moderate-intensity activity may benefit from assessing and reducing barriers specific to walking.

## Figures and Tables

**Table 1:** Descriptive Statistics and Rotated Factor Loadings for Items on Walking Barriers Survey Instrument

Walking Barriers	Descriptive Statistics n = 305		Factor Loadings n = 225^a^		

	Mean Score^b^ (SD)	% DNA^c^	Factor 1^d^	Factor 2^e^	Factor 3^f^
Restrictive clothing	1.9 (1.1)	5.6	.663	.385	.177
Ruining nice clothing	1.8 (1.0)	4.6	.878	.118	-.066
Ruining hairstyle	1.6 (0.9)	5.3	.775	.010	.176
Perspiring	2.1 (1.1)	3.0	.747	.320	.025
Uncomfortable shoes	2.2 (1.2)	3.3	.299	.704	.165
Foot pain	1.6 (0.9)	11.5	.012	.780	.076
Blisters	1.5 (0.9)	11.8	.273	.702	.004
A lot to carry	2.5 (1.1)	4.3	.370	.123	.647
Lack of time	3.2 (1.0)	3.0	.071	−.098	.816
Lack of sidewalks	1.3 (0.7)	13.8	−.118	.287	.594

a Listwise deletion of missing values.

b Participants responded to each potential barrier using a 4-point scale, with 1 indicating *not at all* and 4 indicating *a great deal*.

c Indicates percentage of respondents reporting *does not apply*

d Factor 1 had an eigenvalue of 3.61 with 36.1% of the variance explained.

e Factor 2 had an eigenvalue of 1.36 with 13.6% of the variance explained.

f Factor 3 had an eigenvalue of 1.22 with 12.2% of the variance explained.

**Table 2:** Bivariate Correlations Between Walking Barriers, Vigorous Exercise Barriers, and Moderate-Intensity Physical Activity^a^

	**Variables**					

	**1** ***r*** ***P*** **n**	**2** ***r*** ***P*** **n**	**3** ***r*** ***P*** **n**	**4** ***r*** ***P*** **n**	**5** ***r*** ***P*** **n**	**6** ***r*** ***P*** **n**
**1. Appearance (w)** Mean (SD) = 1.8 (0.8)	—	—	—	—	—	—
**2. Footwear (w)** Mean (SD) = 1.8 (0.8)	.497 <.001 280	—	—	—	—	—
**3. Situational (w)** Mean (SD) = 2.3 (0.7)	.316 <.001 280	.256 <.001 254	—	—	—	—
**4. Internal (v)** Mean (SD) = 2.6 (0.8)	.285 <.001 280	.311 <.001 254	.179 .01 254	—	—	—
**5. External (v)** Mean (SD) = 2.5 (0.8)	.254 <.001 280	.230 <.001 254	.205 .001 254	.614 <.001 295	—	—
**6. Moderate physical activity^b^** Mean (SD) = 3.0 (2.0)	−.272 .02 280	−.034 .78 254	−.341 .003 254	−.171 .12 295	−.299 .01 300	—

W indicates walking barriers; v, vigorous exercise barriers.

a Walking barriers were assessed using a 4-point scale, from 1 = *not at all* to 4 = *a great deal*; vigorous exercise barriers were assessed using a 5-point scale, from 1 = *not at all* to 5 = *a great deal*.

b Percentage of time spent in moderate-intensity physical activity; for ease of interpretation, the nontransformed mean is presented in the table.

**Table 3:** Mean Responses^a^ to Survey About Walking Barriers for Adults Who Engage in Various Types of Walking and Those Who Do Not

**Type of Walking**	**Walking Barriers^b^**					

	**Footwear**		**Situational**		**Appearance**	

	**Mean (SD)**	***P***	** Mean (SD)**	***P***	**Mean (SD)**	***P***
**Stairs instead of elevator**						
Yes	1.7 (0.8)	.28	2.2 (0.7)	.04	1.8 (0.8)	.03
No	1.9 (0.8)		2.5 (0.7)		2.1 (0.0)	
**Walk instead of drive**						
Yes	1.7 (0.8)	.48	2.2 (0.7)	.21	1.7 (0.8)	.01
No	1.8 (0.8)		2.4 (0.7)		2.0 (0.8)	
**Park car away from destination**						
Yes	1.8 (0.8)	.60	2.3 (0.7)	.88	1.8 (0.8)	.78
No	1.8 (0.8)		2.3 (0.7)		1.8 (0.8)	
**Walk at lunch**						
Yes	1.8 (0.7)	.90	2.1 (0.8)	.02	1.8 (0.7)	.39
No	1.8 (0.8)		2.4 (0.7)		1.9 (0.8)	
**Get off bus early**						
Yes	1.8 (0.7)	.93	2.2 (0.8)	.58	2.0 (1.0)	.51
No	1.8 (0.8)		2.3 (0.7)		1.8 (0.8)	
**Extra walking**						
Yes	1.9 (0.8)	.01	2.2 (0.7)	.13	1.9 (0.8)	.64
No	1.6 (0.7)		2.4 (0.7)		1.8 (0.8)	
**Walk for transportation^c^**						
Yes	1.7 (0.8)	.87	2.2 (0.7)	.045	1.9 (0.8)	.33
No	1.8 (0.8)		2.4 (0.7)		1.8 (0.8)	
**Walk for recreation^c^**						
Yes	1.8 (0.8)	.72	2.4 (0.8)	.28	1.7 (0.7)	.054
No	1.7 (0.8)		2.3 (0.7)		1.9 (0.8)	

a Walking barriers were assessed using a 4-point scale, from 1 = *not at all* to 4 = *a great deal*; vigorous exercise barriers were assessed using a 5-point scale, from 1 = *not at all *to 5 = *a great deal*.

b *P* values determined by *t* tests.

c Measured by 3-Day Physical Activity Recall.
